# Highlight: Birds Take tRNA Efficiency to New Heights

**DOI:** 10.1093/gbe/evab045

**Published:** 2021-04-11

**Authors:** Casey McGrath

Birds have been shaped by evolution in many ways that have made them distinct from their vertebrate cousins. Over millions of years of evolution, our feathered friends have taken to the skies, accompanied by unique changes to their skeleton, musculature, respiration, and even reproductive systems. Recent genomic analyses have identified another unique aspect of the avian lineage: streamlined genomes. Although bird genomes contain roughly the same number of protein-coding genes as other vertebrates, their genomes are smaller, containing less noncoding DNA. Scientists are still exploring the potential consequences of this genome reduction on bird biology. In a new article in *Genome Biology and Evolution* titled “Genome size reduction and transposon activity impact tRNA gene diversity while ensuring translational stability in birds,” Claudia Kutter and her colleagues reveal that, in addition to fewer protein-coding genes, bird genomes also contain surprisingly few tRNA genes, while nonetheless exhibiting the same tRNA usage patterns as other vertebrates (Ottenburghs et al. 2021). As tRNAs are a pivotal part of the cellular machinery that translates messenger RNA (mRNA) into protein, this suggests that birds have evolved to use their limited tRNA repertoire more efficiently.

There are many factors that go into maintaining a balanced pool of tRNAs to ensure efficient translation of proteins. Although there are theoretically 64 possible anticodons—three-nucleotide sequences that base-pair with mRNA codons during translation—there are only 20 standard amino acids, meaning that there are generally tRNAs with different anticodons that bind to the same amino acid, referred to as an isoacceptor family. Moreover, the geometry of pairing between the nucleotides in the third position of the codon and anticodon allows for “wobble” base-pairing, enabling a single tRNA anticodon to bind to multiple codons. tRNA genes, even within the same isoacceptor family, can also differ in their sequences, transcription rates, and translation efficiency. Due to all of these factors, ensuring adequate levels of various tRNA molecules for efficient translation in a cell is a complex biological problem.

To investigate the different ways this problem has been solved across vertebrates, Kutter and her co-authors from the Karolinska Institute and Uppsala University in Sweden—including post doc Jente Ottenburghs, graduate student Keyi Geng, and assistant professor at Uppsala University Alexander Suh—undertook the first comprehensive overview of tRNA diversity and evolution in vertebrates. (Ottenburghs has written about their study, including the surprising way this collaboration came about, in a post on his blog, Avian Hybrids.) Based on their findings in mammals, the authors extrapolated a uniform set of 500 tRNA genes that they expected to remain relatively consistent across lineages. However, when they added 55 avian genomes to their study, representing all of the major bird lineages, they came across some unexpected findings: “To our surprise, there was much more divergence across vertebrates than we expected to find,” says Kutter. In particular, bird genomes stood out, containing an average of just 169 tRNA genes compared with 466 in reptiles, 579 in mammals, 813 in fish, and 1,229 in amphibians. According to Kutter, “While ongoing genome assembly efforts had shown that bird genomes have a smaller genome size, we were not expecting that this would also affect tRNA genes, since tRNA gene redundancy ensures that enough tRNA molecules are transcribed for efficient mRNA translation.”

This led the authors to a new question: What did this contraction in tRNAs mean for translation in birds? Although tRNA gene number and complexity were greatly reduced compared with other vertebrates, in general, the authors found that preferences for certain isoacceptor families were in line with the wobble pairing strategies observed in other eukaryotic genomes, suggesting that tRNA gene usage in birds follows the overall codon usage seen in vertebrates. This led the researchers to posit that the functional constraints on tRNAs seen in other vertebrates were maintained during early avian evolution: “Despite this decrease [in tRNAs] millions of years ago, the pool of tRNA anticodons and mRNA codons is still balanced across bird species to ensure optimal translational efficiencies.” 

**Fig. 1:**
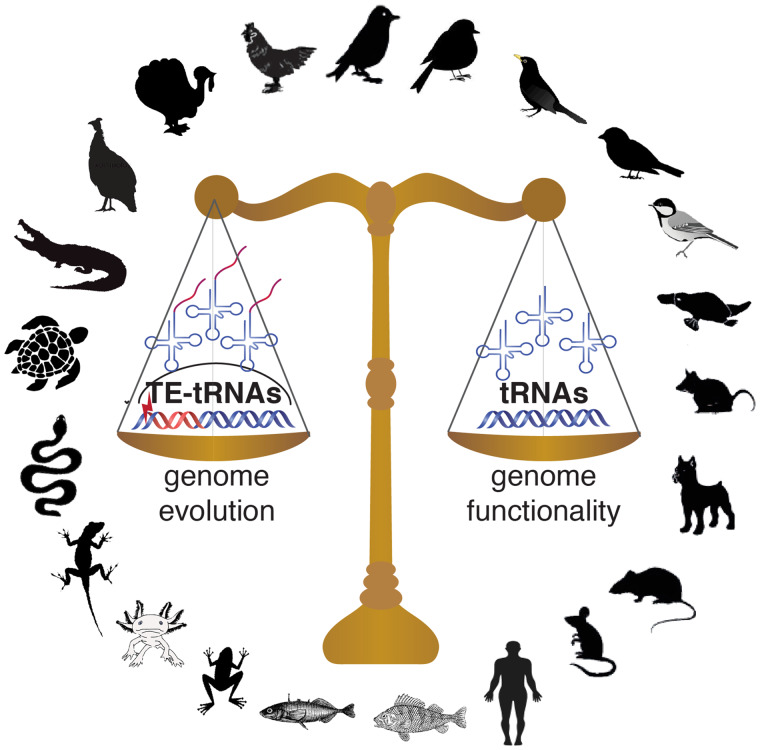
The tRNA repertoires of vertebrate genomes reflect a balance between translational efficiency, genome functionality, and the emergence of new tRNA copies via transposable element-mediated activity.

Another surprising element to the study was the impact of transposable elements on the repertoire of tRNA genes in avian genomes. In some bird genomes studied, in addition to finding functional tRNA genes, Ottenburghs et al. (2021) identified hundreds, sometimes thousands, of tRNA-like sequences embedded in transposable elements. Although most transposable elements are silenced by epigenetic control mechanisms and become nonfunctional, some may remain active and create new regulatory roles through their mobilization. Through multiplication of transposable elements, the embedded tRNA-like sequences may be carried to new genomic locations, where selection will constrain the tRNA gene sequence, whereas the accompanying transposable element sequence may erode. This process contributes to shaping the pool of available tRNA genes in some avian genomes, adding another layer of complexity to tRNA gene evolution in this vertebrate lineage (fig. 1).

The lesson here, according to Kutter, is that “we should reconsider our current assumptions of gene functionality with regards to redundancy.” To explore this idea further, Kutter plans to expand this work to include better mechanistic insight and more functional genomic studies. “It would be insightful to include more species and look deeper into branches, as well as investigate snake genomes and the early vertebrate radiation. Performing studies that go beyond current model organisms may reveal even more unexpected findings.” One limitation of these potential studies is that they will require access to high-quality genome assemblies and resources, such as tissue samples from different developmental time points and suitable reagents like antibodies that work across species. Kutter believes these efforts will pay off in the long run however: “Our work has shown us yet again that evolution still has a lot of surprises up its sleeves, and we can get a glimpse into these by looking beyond model organisms.”
